# PMN-MDSCs Enhance CTC Metastatic Properties through Reciprocal Interactions via ROS/Notch/Nodal Signaling

**DOI:** 10.3390/ijms20081916

**Published:** 2019-04-18

**Authors:** Marc L. Sprouse, Thomas Welte, Debasish Boral, Haowen N. Liu, Wei Yin, Monika Vishnoi, Debalina Goswami-Sewell, Lili Li, Guangsheng Pei, Peilin Jia, Isabella C. Glitza-Oliva, Dario Marchetti

**Affiliations:** 1 Biomarker Research Program Center, Houston Methodist Research Institute, Houston, TX 77030, USA; Msprouse@houstonmethodist.org (M.L.S.); twelte@houstonmethodist.org (T.W.); dboral@houstonmethodist.org (D.B.); hnliu@houstonmethodist.org (H.N.L.); Weiyin9999@yahoo.com (W.Y.); mvishnoi@houstonmethodist.org (M.V.); goswami.rima1985@gmail.com (D.G.-S.); Lielectric@gmail.com (L.L.); 2 Center for Precision Health, School of Biomedical Informatics, University of Texas Health Science Center at Houston, Houston, TX 77030, USA; Guangsheng.Pei@uth.tmc.edu (G.P.); Peilin.Jia@uth.tmc.edu (P.J.); 3 Department of Melanoma Medical Oncology, UT MD Anderson Cancer Center, Houston, TX 77030, USA; ICGlitza@mdanderson.org; 4 Institute for Academic Medicine, Houston Methodist Hospital, Houston, TX 77030, USA

**Keywords:** circulating tumor cells (CTCs), polymorphonuclear-myeloid derived suppressor cells (PMN-MDSCs), Heterotypic CTC clusters, mutual CTC/PMN-MDSC activation cycle, biomarkers and signaling pathways, breast cancer, melanoma

## Abstract

Intratumoral infiltration of myeloid-derived suppressor cells (MDSCs) is known to promote neoplastic growth by inhibiting the tumoricidal activity of T cells. However, direct interactions between patient-derived MDSCs and circulating tumors cells (CTCs) within the microenvironment of blood remain unexplored. Dissecting interplays between CTCs and circulatory MDSCs by heterotypic CTC/MDSC clustering is critical as a key mechanism to promote CTC survival and sustain the metastatic process. We characterized CTCs and polymorphonuclear-MDSCs (PMN-MDSCs) isolated in parallel from peripheral blood of metastatic melanoma and breast cancer patients by multi-parametric flow cytometry. Transplantation of both cell populations in the systemic circulation of mice revealed significantly enhanced dissemination and metastasis in mice co-injected with CTCs and PMN-MDSCs compared to mice injected with CTCs or MDSCs alone. Notably, CTC/PMN-MDSC clusters were detected in vitro and in vivo either in patients’ blood or by longitudinal monitoring of blood from animals. This was coupled with in vitro co-culturing of cell populations, demonstrating that CTCs formed physical clusters with PMN-MDSCs; and induced their pro-tumorigenic differentiation through paracrine Nodal signaling, augmenting the production of reactive oxygen species (ROS) by PMN-MDSCs. These findings were validated by detecting significantly higher Nodal and ROS levels in blood of cancer patients in the presence of naïve, heterotypic CTC/PMN-MDSC clusters. Augmented PMN-MDSC ROS upregulated Notch1 receptor expression in CTCs through the ROS-NRF2-ARE axis, thus priming CTCs to respond to ligand-mediated (Jagged1) Notch activation. Jagged1-expressing PMN-MDSCs contributed to enhanced Notch activation in CTCs by engagement of Notch1 receptor. The reciprocity of CTC/PMN-MDSC bi-directional paracrine interactions and signaling was functionally validated in inhibitor-based analyses, demonstrating that combined Nodal and ROS inhibition abrogated CTC/PMN-MDSC interactions and led to a reduction of CTC survival and proliferation. This study provides seminal evidence showing that PMN-MDSCs, additive to their immuno-suppressive roles, directly interact with CTCs and promote their dissemination and metastatic potency. Targeting CTC/PMN-MDSC heterotypic clusters and associated crosstalks can therefore represent a novel therapeutic avenue for limiting hematogenous spread of metastatic disease.

## 1. Introduction

Transit through the circulatory system imposes a hostile environment on circulating tumor cells (CTCs), which are shed from primary and/or metastatic tumors and known “seeds” of fatal metastatic disease. Previous studies have shown tumor cell-intrinsic strategies that help overcome this constraint by employing programs of epithelial-mesenchymal transition (EMT), cell stemness, or by migrating as two or more aggregated CTCs in blood of cancer patients (CTC clusters) [1]. Reports in multiple cancer types have also suggested a link between CTC clusters presence and worse clinical outcome [1]. Whether non-tumor, myeloid-derived suppressor cells (MDSCs) could participate in CTC clusters is largely unknown, because CTC enrichment by lymphocyte (CD45) depletion was used in major studies with intention to observe CTC clusters [2,3]. Conversely, tumor cells are well known for their ability to interact with normal cells, modulate their activities and thrive in this microenvironment. It has also been established that peripheral mononuclear (PMN) cell subsets expand in blood of cancer patients. Under the impact of tumor-cell derived signals, PMNs are at a phenotypically “immature” developmental stage and display inhibitory function in T cell activation [4]. Accordingly, these cells are designated PMN myeloid-derived suppressor cells (PMN-MDSCs). Many reports have demonstrated pro-tumorigenic roles of PMN-MDSCs at multiple levels and stages of metastasis, including the suppression of the adaptive immune response, effects on tumor angiogenesis and cancer stem cell properties, and shaping the generation of pre-metastatic niches (reviewed in [4,5,6,7]). Despite the important roles played by MDSCs altering tumor cell-initiating competence and providing pre-metastatic niches [7,8,9,10], mechanisms underlying direct effects of circulatory PMN-MDSCs on CTCs are not understood.

An important characteristic of PMN-MDSCs is the production of reactive oxygen species (ROS) [4,5,6,7]. ROS signaling has a hormetic effect in cancer cells: below genotoxic levels, ROS are mitogenic and stimulate cell proliferation whereas higher ROS levels result in cell-cycle arrest through the activation of DNA damage response [11,12]. A well-characterized mechanism of mitogenic ROS signaling involves the redox sensitive enzyme phosphatase and tensin homolog (PTEN) which loses its activity upon oxidation by H_2_O_2_ [13,14]. Therefore, ROS activates the PI3K/Akt/mTOR survival pathway through the reversible oxidation of tumor suppressor PTEN [15]. A recent study on melanoma resistant to BRAF/MEK inhibitors revealed that ROS levels increase at advanced, drug-resistant stages and provide direct benefits on tumor cell survival/proliferation [16]. One tumor cell growth-promoting mechanism is through the crosstalk between NRF2 (also known as NFE2L2) cytoprotective transcription factor and the Notch pathway [17]. Conversely, Notch signaling is known to regulate tumor cell stemness [17], and we reported Notch functionalities in CTC subsets that are brain metastasis-competent [3,18]. Altogether, these findings raise the question as to whether PMN-MDSCs could have a dual role: signaling to CTCs via diffusible ROS and Notch ligand/Notch signaling pathway by cell–cell contact dependency.

Notably, CTC transcriptomic analyses performed in our laboratory indicated highest expression of Nodal [3], an embryonic morphogen of the transforming growth factor-beta (TGF-β) superfamily, shown to be expressed in aggressive cancers, e.g., triple negative breast and melanoma cancers, supporting tumor progression and resistance to conventional chemotherapies [19,20,21]. Nodal is a target gene of Notch signaling via two NICD/CSL target sites present in its promoter [20]. Nodal expression facilitates cell plasticity which is an important characteristic throughout the different steps of the metastasis process. Nodal expression has been recently shown to promote the M2, e.g., “type 2” phenotype of monocytic innate immune cells [21]. This “type 2” phenotype is pro-tumorigenic and characterized by enhanced ROS production in the PMN lineage [4,5,6,7]. Here, we tested the hypothesis that patient-isolated circulating PMN-MDSCs directly interact with CTCs, induce pro-survival CTC pathways via ROS and the Notch signaling pathway, with their pro-tumor functions being modulated by CTC-derived Nodal expression.

## 2. Results

### 2.1. Isolation and Characterization of CTC/PMN-MDSC Clusters

**Figure 1:**
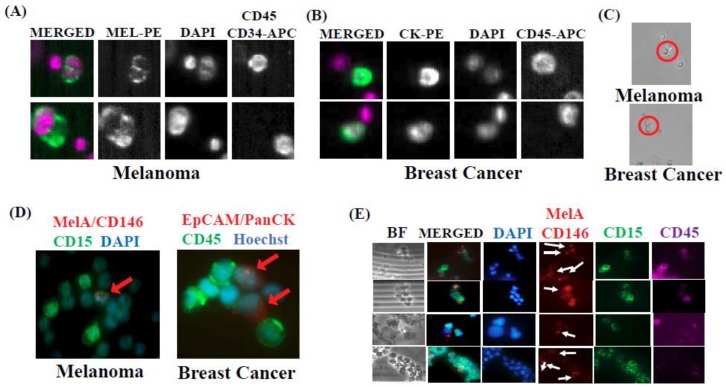
(**A**,**B**) The detection of heterotypic CTC clusters using CellSearch^®^ analyses of blood obtained from melanoma or breast cancer patients, respectively. (**C**) Representative images capturing a two-cell heterotypic cluster between one CTC and one cell of the myeloid lineage (top), and a homotypic CTC/CTC cluster (bottom) using CellSieve™ cell filtration device. (**D**) Detection of large heterotypic CTC clusters using the Parsortix^®^ filtration device from blood of melanoma and breast cancer patients. Representative images are shown. Red arrows point out to MelA/CD146-positive cells (melanoma CTCs) or EpCAM/PanCK-positive cells (breast cancer CTCs), respectively. (**E**) Representative images of melanoma patient CTC/PMN-MDSC heterotypic clusters captured by Parsortix^®^ microfluidic device. Heterotypic clusters between FACS-sorted Lin−/CD45−/MelA+/CD146+ cells (melanoma CTCs) and Lin+/CD45+/CD33+/CD15+ cells (melanoma PMN-MDSCs) from a representative patient are shown. White arrows point to CTCs. Scale bar = 20 μm.

To elucidate interplays between CTCs and circulatory MDSCs, we used multi-parametric fluorescence activated cell sorting (FACS) to isolate putative CTCs and PMN-MDSCs from peripheral blood of patients diagnosed with metastatic breast and melanoma cancers (*n* = 15 and *n* = 18, respectively; the clinical-pathological parameters of patients enrolled in this study are provided) (Figures S1 and S2). CTCs and PMN-MDSCs were isolated in parallel from peripheral blood mononuclear cells (PBMCs) of the same patient sample. We used gating strategies previously reported to be successful for the isolation of putative CTCs [3,22,23]; along with FACS selection to obtain pure populations of PMN-MDSCs by employing established biomarkers for these cells [4,5,6,7,8]. Following red blood cell lysis, PBMCs were subjected to FACS and underwent doublet discrimination and dead cell elimination. Next, the CD45+ cell population was positively selected for CD33+/CD11b+, followed by CD14− and CD15+ selection to isolate PMN-MDSCs [4,5,6]. Conversely, the CD45− cell population was subjected to further depletion of “normal” cells using lineage-specific antibodies (CD34+/CD73+/CD90+/CD105+) to obtain lineage-negative (Lin-) cells. This was followed by the positive selection for either PanCK+ cells (breast cancer CTCs) [3] or CD146+/Melan-A+ cells (melanoma CTCs) [18]. Correct cell-surface biomarker selection and FACS procedures to isolate PMN-MDSCs and CTCs were confirmed by immunocytochemistry. Importantly, the presence of heterotypic CTC/PMN-MDSC clusters was detected with clusters captured by using either the FDA-cleared CellSearch^®^ CTC platform (Menarini Silicon Biosystems, Huntington Valley, PA, USA; capture of heterotypic clusters from patient’s peripheral blood was achieved according to platform specifications) [3,22,24], or by cell filtration/microfluidic devices, e.g., Parsortix^®^ (Angle Inc, Guildford, UK) (www.ANGLE.com) [25] and CellSieve™ (Creatv MicroTech, Potomac, MD, USA); coupled with high-definition immunofluorescence microscopy (Figure 1) [3].

**Figure 2:**
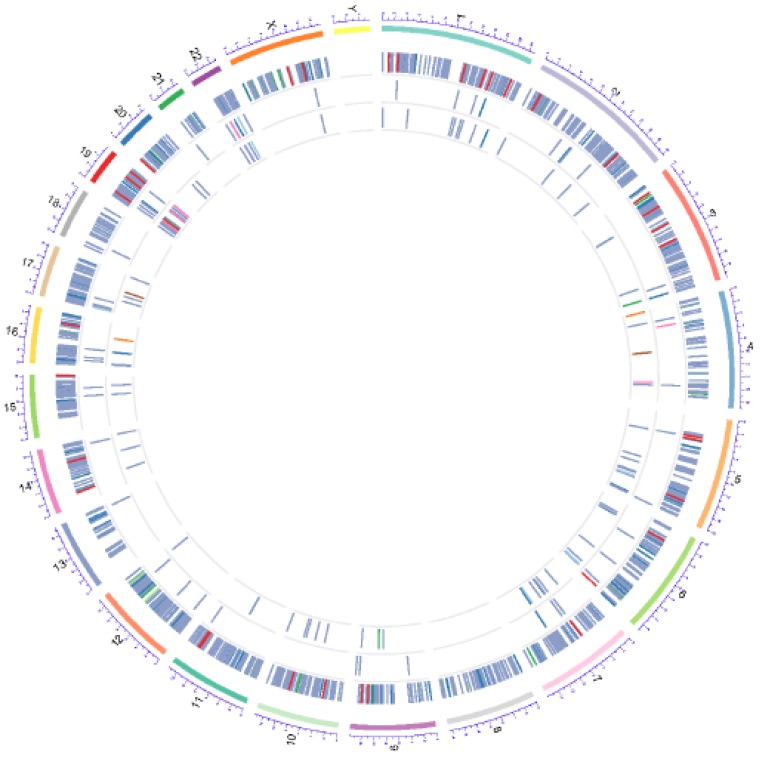
Circos plot of the somatic genetic variants detectable in Lin-/DAPI+/EpCAM+/CK+ CTCs (outer layer), and Lin+/DAPI+/CD14+ (middle layer), and Lin+/DAPI+/CD15+ (inner layer) cell populations selected from blood of a metastatic breast cancer patient via multi-parametric FACS.

Although previous studies have described the presence of circulating tumor aggregates of CTCs with non-tumor cells [26], none have shown CTCs clustered with MDSCs. Furthermore, mechanisms that provide CTC clusters survival and metastatic advantages while transiting in the vasculature are not well understood [27,28]. Using CellSearch^®^-based blood analyses, we detected CTC clusters in 50% of breast or melanoma cancer patients (16 out of 33). Conversely, CTC clusters could be captured in 100% of patients analyzed by Parsortix^®^ or other cell filtration devices, e.g., CellSieve™ (Figure 1). Notably, we observed heterotypic interactions between CTCs and non-tumor cells (heterotypic CTC clusters) in 6 out of 8 patients analyzed by CellSearch^®^, at a frequency of 1–5 clusters per 7.5 mL blood, the certified blood volume for CellSearch^®^ clinical CTC testing [24]. To confirm CellSearch^®^ findings, we used the Parsortix^®^ cell-separation system to capture individual CTCs and CTC clusters directly from patients’ blood. Immunofluorescent (IF) staining of cells captured by Parsortix^®^, showed heterotypic clusters composed of CTCs and PBMCs, the latter as CD45+ cells. These heterotypic clusters were detected in melanoma (Melanoma CTCs: CD45−/CD34−/CD90−/CD105−/CD73− but CD146+/MelA+ cells), as well as in breast cancer patients’ blood (Breast cancer CTCs: EpCAM+/PanCK+/DAPI+ but CD45− cells) [22]. Ratios between CTCs and CD45+ cells in these clusters were in the 1:1 to 1:4 range. Additional IF staining demonstrated the presence of CD45+/CD33+/CD15+ cells (part of the myeloid lineage) as the key component of heterotypic CTC clusters (Figure 1). Next, genomic characterization was performed to validate the cellular content of heterotypic clusters containing putative breast cancer CTCs and MDSCs. Whole exome sequencing was performed for these cell subpopulations isolated by FACS with corresponding cell markers. Somatic mutations were defined in reference to sequencing analyses of Lin-positive (CD45+/CD34+/CD105+/CD90+/CD73+ cells) vs. Lin-negative populations. Whole exome sequencing (WES) detected multiple somatic variants in CTCs; some were in chromatin modeling factors KAT6B, KMT2D and KDM6A genes which are frequently mutated in breast cancer. Surprisingly, somatic variants were also detected in MDSCs (CD45+/CD33+/CD11b+, and CD14+ or CD15+ MDSC cell sub-populations), although at a much lower frequency compared to CTCs. The mutational patterns of CD14+ and CD15+ cells were also distinct, suggesting different mutagenic events occurring for the two MDSC subpopulations (Figure 2).

### 2.2. CTC-Driven Metastatic Dissemination is Enhanced by PMN-MDSCs

**Figure 3:**
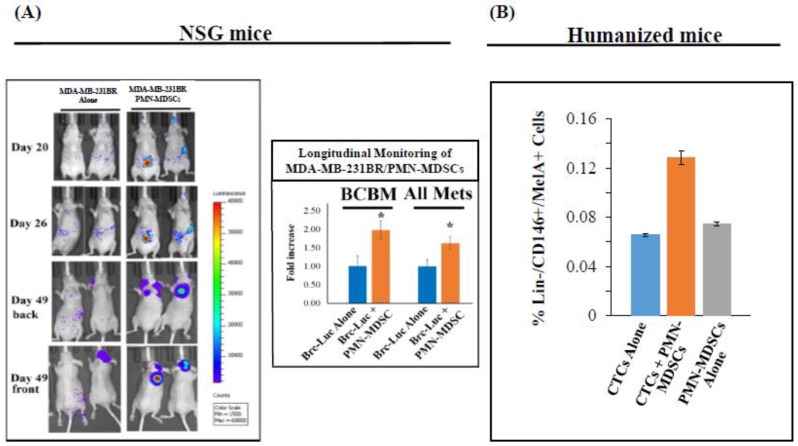
Metastatic competence of CTCs is significantly enhanced by co-transplantation of PMN-MDSCs isolated from the same patient in animals. (**A**) Representative images of mice injected intracardiacally with luciferase-labeled MDA-MB-231BR cells (Brc-Luc) cells, either alone or with unlabeled PMN-MDSCs (1.0 × 10^3^ Brc-Luc cells vs. 5.0 × 10^5^ PMN-MDSCs), followed by bioluminescence imaging at indicated times. Bioluminescence measurements of metastasis/BCBM per treatment of mice populations (*n* = 10/subgroup; * *p* < 0.05). (**B**) Detection of FACS-selected patient-derived CTCs (Lin−/DAPI+/CD146+/MelA+ cells) and PMN-MDSCs (Lin-/DAPI+/CD33+/CD15+ cells) obtained from blood of a representative melanoma patient injected in humanized mice, either alone (CTC Alone/PMN-MDSC Alone), or together (CTC + PMN-MDSC) (1.0 × 10^3^ CTCs, vs. 5.0 × 10^5^ PMN-MDSCs). Data show the percentage of live human melanoma CTCs (Lin−/CD146+/MelA+ cells) isolated from humanized murine blood 22 weeks following cell injection. Data represent the mean ± SD of six independent experiments (*n* = 6).

Next, we co-injected luciferase-labeled breast cancer MDA-MB-231BR (Brc-luc) cells, along with patient-matched PMN-MDSCs into the systemic circulation of immunodeficient NOD-SCID-gamma (NSG) mice by intracardiac (i.c.) (left ventricular) injection. Mice injected with either Brc-luc cells or PMN-MDSCs alone were used as controls. Mice were euthanized at 4 weeks after cell injection and target organs were examined for evidence of metastasis. Luciferase quantitation/IVIS imager analyses detected the presence of significantly higher number of metastasis in organs of mice co-injected with Brc-luc cells and PMN-MDSCs compared to mice injected with Brc-luc cells alone (Figure 3). Interestingly, despite the short life-span of PMN-MDSCs (1–2 weeks), we detected heterotypic clusters in the peripheral blood and bone marrow of NSG mice co-injected with Brc-luc cells and PMN-MDSCs for significantly longer periods, compared to mice injected with Brc-luc cells or PMN-MDSCs alone. To determine whether the presence of a functional immune system affected these metastatic patterns, we repeated experiments employing humanized NSG mice (CD34+ human placental stromal cell (HPSC)-injected) [29] as immunocompetent mouse model, and injection of melanoma Lin-negative/CTC-enriched cell populations [22]. We obtained similar results without detecting any significant differences either in micro-metastatic foci counts or prolonged CTC survival in immunocompetent (humanized) vs. immunodeficient (NSG) animals (Figure 3). This implies that concurrent CTC and PMN-MDSC cell injection in the same animal induced spontaneous distant metastasis independent of PMN-MDSC suppressive effects on adaptive and NK cell antitumor activities. Results also suggest that enhanced CTC survival and metastatic dissemination is mediated through PMN-MDSCs “priming” CTCs, with the former affecting properties of the latter. Altogether, these findings introduce notions that PMN-MDSC actions on CTCs are alternative to and/or distinct from the established immunosuppressive roles of PMN-MDSCs on T cells [4].

**Figure 4:**
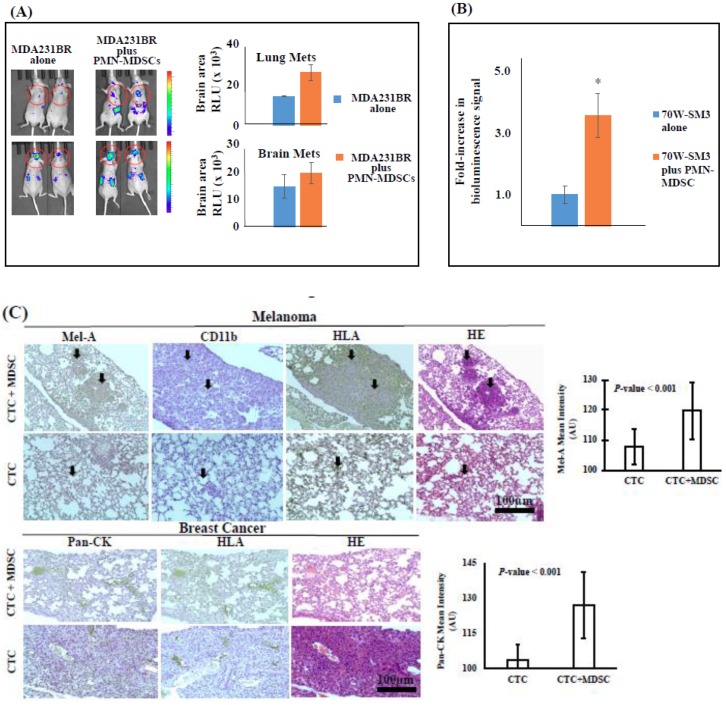
The detection of bioluminescence signal following the injection of human brain-metastatic breast cancer (MDA-MB-231BR) (**A**) (red circles refer to the detection of brain metastasis in mice) or melanoma (70W-SM3) mice (**B**) cells with or without co-transplantation with circulatory PMN-MDSCs isolated from blood of respective patients. Representative animals are shown (*n* = 3/subgroup; * *p* < 0.001). (**C**) CTC metastatic competence is increased by PMN-MDSCs. Patient-isolated CTCs were injected together with PMN-MDSCs derived from respective breast cancer or melanoma patient blood via intra-cardiac route. Following animal euthanization and tissue organ processing, tumor cells were detected in metastatic foci by histopathology staining using human tumor cell-specific markers as indicated. Animal lung tissues of four breast cancer or melanoma patients were analyzed following the injection of CTCs/PMN-MDSCs. Magnified images (100×) were captured and quantified by free-access Image J software using the reciprocal intensity (250-*y*) method [22]. Controls consisted of detection of human tumor cells in tissues of animals injected only with CTCs. Tissues of animals injected only with patients’ MDSCs stained negative for human tumor cell-specific biomarkers (data not shown).

To authenticate the long-term outcome of interactions between CTCs and PMN-MDSCs, we co-injected luciferase-labeled, highly brain-metastatic breast cancer (Luc-MDA-MB-231BR) or melanoma (Luc-70W-SM3) cells with patient-isolated PMN-MDSCs into the systemic circulation of NSG mice, and measured luciferase activity/metastasis at target organs using IVIS imager. We detected a marked increase in tumor cell sequestration to the bone marrow and dissemination to lungs and brains as early as 3 h post-injection of tumor cell/PMN-MDSC clusters compared to tumor cells alone (Figure 4). This was recapitulated on subsequent increased luciferase signal measured in live animals on days 6, 26 and 52 post-injection, as well as by ex vivo imaging of organs harvested after mice euthanization (Figure 4). Findings were validated by histopathological and immunohistochemical (IHC) analyses showing a significantly increased presence of human tumor cells (HLA+/MelA+ melanoma or HLA+/PanCK+ breast cancer) in lung tissues of mice injected with CTCs together with PMN-MDSCs vs. CTCs alone, isolated in parallel either from melanoma or breast cancer patients’ blood (Figure 4).

### 2.3. High Number of Circulatory PMN-MDSCs and Plasma ROS Levels are Linked to Metastasis

**Figure 5:**
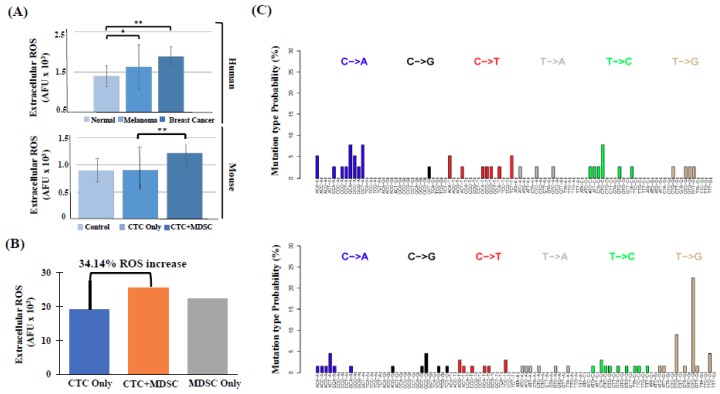
(**A**) The detection of significantly higher exogenous ROS levels in serum of breast cancer or melanoma patients compared to healthy donors (Normal) (*n* = 4; * *p* < 0.01; **p < 0.001) (Upper panel); or in serum of animals previously (6 days) injected with CTCs + PMN-MDSCs directly isolated from patients’ blood in parallel vs. CTC alone vs. no cell injection (Control) (*n* = 4; ** *p* < 0.001). (**B**) CTC/PMN-MDSC co-cultures contributed to significantly higher extracellular ROS (~35% increase; *p* < 0.001). CTCs/PMN-MDSCs from co-cultures were injected in mice and ROS levels compared to ones from mice injected with either CTCs or MDSCs alone (*n* = 8/subgroup). (**C**) Mutational signatures of M-MDSC (Lin+/DAPI+/CD33+/CD14+ (upper panel)) and PMN-MDSC (Lin+/DAPI+/CD33+CD15+ (lower panel)) cell populations by WES analyses.

The iNOS-mediated ROS production is a fundamental function of PMN-MDSCs [4,5,6,7]. Because we detected a positive correlation between high numbers of MDSCs and metastatic incidence in melanoma and breast cancer patients, we measured plasma ROS levels in these patients. We found that melanoma patients had 16.5% higher (* *p* = 0.02), while breast cancer patients had 49.1% higher (** *p* < 0.001) levels of exogenous ROS compared to healthy donors (control) (Figure 5A). Furthermore, elevated ROS levels found in these patients correlated with high numbers of “type 2” PMN-MDSC cells (R = 0.90; *p* < 0.001). To confirm that the cellular source of elevated ROS in cancer patients belongs to the MDSC lineage, we compared the amount of exogenous ROS produced among MDSC subtypes in vitro and found that PMN-MDSCs generated nearly double (1.8-fold; *p* < 0.001) the amount of ROS compared to other MDSC subtypes. Additionally, mice that were co-injected with CTCs and PMN-MDSCs exhibited strikingly augmented (~35% increase) ROS levels than mice injected with CTCs alone (Figure 5A). Similarly, a ~34% increase of exogenous ROS was observed in vitro when patient-derived CTCs and MDSCs were co-cultured together (72 h) compared to CTCs or MDSCs alone (Figure 5B). ROS is known to cause DNA damage by producing 8-hydroxyguanine (^8-OH^G), which can be paired with Adenosine [30]. Misreading or incorporation of ^8-OH^G during DNA replication results in two types of substitutions: G > T (C > A on reverse strand) or A > C (T > G on the reverse strand). Because chances of incorporation of free ^8-OH^G during DNA replication is increased in the presence of high intracellular ROS production, the relative frequency of these mutations can be used as a surrogate marker to assess the level of ROS production in different cell types. Accordingly, we performed whole exome sequencing (WES) of breast cancer patient-derived CD15+ PMN-MDSCs and CD14+ M-MDSCs to assess differences in ROS-associated mutation signatures. Though we detected both C > A and T > G mutation signatures in PMN-MDSC and M-MDSC cell populations, the presence of T > G substitutions were significantly more frequent in PMN-MDSCs (Figure 5C), suggesting that PMN-MDSCs contribute to the elevated ROS levels detected in patients’ peripheral blood.

### 2.4. PMN-MDSCs Form Clusters with CTCs and Promote CTC Proliferation

**Figure 6:**
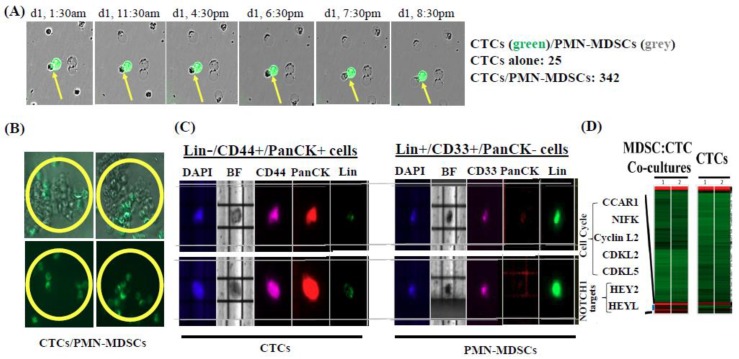
Patient-isolated PMN-MDSCs interact with and promote CTC proliferation. (**A**) CTCs and PMN-MDSCs isolated from blood of BCBM patients were co-cultured over time and their growth was monitored by IncuCyte^®^ S3 Live-Cell Analysis System. Snap-shots were taken at indicated times using IncuCyte^®^ real-time videomicroscopy. Yellow arrows point out to specific CTC/PMN-MDSC interactions. (**B**) Heterotypic clusters formation between CTCs and PMN-MDSCs detected over time (>72 h) by IncuCyte^®^ following CTC/PMN-MDSC co-culturing. CTCs were labeled green using Vybrant™ DiO cell-labeling solution (Molecular Probes, Inc., Eugene, OR, USA), and clusters were visualized (yellow circles) and analyzed for CTCs and PMN-MDSCs presence. A >10-fold multiplication of CTC numbers could be detected when clustered with PMN-MDSCs. (**C**) CTC/PMN-MDSC clusters were retrieved from co-cultures employing CellCelector™ (ALS, Inc., Jena, Germany), dissociated into single-cell suspensions, stained for specific markers, and analyzed by the DEPArray. Representative images of single CTCs and PMN-MDSCs from the same patient are shown. BF = Brightfield image. (**D**) Transcriptomic analyses of CTCs retrieved from CTC/PMN-MDSC clusters following co-cultures vs. CTCs alone. Highest expression of genes related to cell cycle progression and Notch1 targets are shown. Dissociated cells from CTC/PMN-MDSC clusters were collected directly into a pre-chilled tube maintained at 4 °C containing RNA lysis buffer. Total RNA was collected according to the manufacturer’s protocol (Macherey-Nagel, Inc., Düren, Germany). Subsequently, RNA and cDNA amplifications, quality controls and gene expression arrays were performed at the Sequencing and Non-coding RNA Program Core (MD Anderson Cancer Center, Houston, TX, USA) using the HTA 2.0 gene chip (Affymetrix, Inc., Santa Clara, CA, USA). Subsequent pathway enrichment analysis was performed using the Ingenuity pathway software (IPA version 01–07; Qiagen, Inc., Hilden, Germany), as previously described [3].

To investigate direct effects of PMN-MDSCs on CTCs, we labeled CTCs with the Vybrant™ DiO dye and co-cultured them with patient-matched PMN-MDSCs isolated from respective patients at a 1:500 ratio (CTC:PMN-MDSC) for up to 72 h. Next, we monitored cellular in vitro growth of CTC/PMN-MDSC co-cultures using the IncuCyte^®^ S3 live-cell imaging system, and detected higher CTC numbers as result of CTC co-culturing with PMN-MDSCs (Figure 6A). We consistently observed the formation in vitro of heterotypic clusters between CTCs and PMN-MDSCs when co-cultured together and persisting over time (Figure 6B). We performed several control experiments to demonstrate the specificity of CTCs and PMN-MDSC interactions. First, these observations were reproduced in vitro employing human melanoma and breast cancer cell lines (70W-SM3/MDA-MB-231BR cells, respectively) co-cultured with PMN-MDSCs derived from disease-matched patients. Second, we co-cultured CTCs with varying amounts of PMN-MDSCs to understand whether heterotypic cluster formation is dependent on defined stoichiometric balances. We found that although the average size of clusters decreased with lower PMN-MDSC inputs (likely due to non-availability of MDSCs), the initial formation of heterotypic CTC/PMN-MDSC clusters remained unaffected. Third, we performed DNaseI treatment of whole blood before the initiation of co-culture experiments to rule out the possibility that cluster formation, e.g., due to aberrant clumping of extra-nuclear material originated from apoptotic cells. DNaseI treatment did not attenuate CTC/PMN-MDSC cluster formation (data not shown).

Next, to determine biological effects of PMN-MDSCs on CTCs, we measured CTC proliferation rates and CTC numbers after co-culturing with PMN-MDSCs using the IncuCyte^®^ S3 live-cell imaging system and Click-iT^®^ EdU imaging kit (ThermoFisher Scientific, Inc., Wlatham, MA, USA). We observed a significant increase in EdU-positive CTCs compared to CTCs cultured alone (Figure 6A). As additional level of scrutiny, viability, biomarker expression and quantitation of CTC/PMN-MDSCs were confirmed by DEPArray analyses of CTC/PMN-MDSC clusters retrieved from co-cultures by the CellCelector™ (ALS, Inc., Jena, Germany), dissociated into single-cell suspensions by trypsinization, and analyzed by DEPArray to obtain single, viable CTCs and PMN-MDSCs. These analyses confirmed that both CTCs and PMN-MDSCs from co-cultures retained their viability and cell surface biomarker definition throughout the process of cluster formation and subsequent dissociation analyses (Figure 6C). To validate these results and their biological significance, we performed DEPArray™ isolation of CTCs from dissociated CTC/PMN-MDSC clusters and subjected them to transcriptomic analyses. CTCs isolated from in vitro heterotypic CTC/PMN-MDSC clusters displayed a significant increase in the expression of proliferation-related (cell-cycle, Notch pathway-associated) genes compared to CTCs cultured alone (Figure 6D). This was also verified by investigating increased rates of positive Ki67 staining (Ki67+ CTCs/Ki67+ PMN-MDSCs) quantified in co-cultured CTCs and PMN-MDSCs by DEPArray™ interrogation: 75% of all CTCs and 66% of all MDSCs analyzed by DEPArray™ were proliferation-competent as Ki67-positive cells (Figure S3). Complementary studies employing melanoma and breast cancer cell lines (70WSM3 and MDA-MB-231BR, respectively) confirmed these results: enhanced cell growth and Ki67 expression positivity were detected when these cells were co-cultured with patient-derived PMN-MDSCs (Figure S3).

### 2.5. PMN-MDSCs Facilitate CTC Survival through Dual Activation of ROS-NRF2-ARE Axis and Notch Signaling Pathways

**Figure 7:**
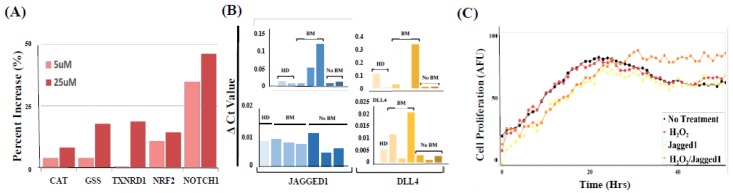
(**A**) Dose-dependent increase of NRF2 target genes in melanoma CTCs following H_2_O_2_ treatment. Data from a representative experiment (*n* = 4) are shown. (**B**) Augmented gene expression of Notch1 ligands Jagged1 and DLL4 in PMN-MDSCs isolated from patients with/without brain metastasis (BM). HD = corresponding analyses of PMN-MDSCs isolated from blood of healthy donors. (**C**) CTC proliferation is up-regulated by the combinatorial treatment of oxidative stress and MDSC Jagged1 measured by real-time IncuCyte^®^ Live-Cell Analysis System in 3D cell conditions. No treatment (negative control; black), H_2_O_2_ (25 μM; red), recombinant human Jagged1 (100 μM; yellow), and combinatorial (orange) are shown. Data are representative of six independent experiments (*n* = 6).

ROS generated from MDSCs is known to provide an immune privileged microenvironment for tumor cells and to promote tumor cell proliferation through the activation of the NRF2-ARE pathway [4,5,6,7]. Because NRF2-ARE upregulates Notch expression in cancer cells [31], we investigated the involvement of Notch in PMN-MDSC—CTC crosstalks. First, we found that treatment of patient-derived melanoma CTCs with hydrogen peroxide (H_2_O_2_) (0–25 µM)—a major component of ROS—caused a dose-dependent increase of NRF2 target gene expression along with downstream anti-oxidant CAT, GSS, and TXNRD1 effector genes (Figure 7A). Second, we observed that CTCs co-cultured with PMN-MDSCs had significantly higher levels of Notch1 gene expression, along with an increase of cell-cycle genes and Notch target genes *HEY1* and *HES1* [17,19]. However, in the presence of the NRF2 inhibitor ML385 (5 µM) [27], PMN-MDSCs could not induce Notch1 gene expression, suggesting that PMN-MDSCs upregulate Notch1 expression in CTCs by the NRF2-ARE axis. We posited that activation of the Notch signaling pathway involves the engagement of Notch1 receptor present on the cell surface of CTCs [3] with concurrent presence of Notch1 ligands Jagged1/DLL in PMN-MDSCs. We detected high levels of Notch1 ligands in PMN-MDSCs isolated from multiple patients (Figure 7B). We also detected signal heterogeneity and distinction of Notch ligands type, gene expression, and according to the brain-metastatic phenotype, although we could not reach statistical significance. However, the outcome of Notch/Notch ligand interactions was corroborated in vitro by augmented proliferation when CTC were exposed to a combination of H_2_O_2_ and Jagged1, compared to treating CTCs with H_2_O_2_ or Jagged1 alone. CTC proliferation, assessed in real-time (Incucyte™) and in 3D cell culture conditions using a modified sphere-forming media for CTC culturing [23], was augmented over time by oxidative stress and Jagged1 combinatorial treatment (Figure 7C).

### 2.6. CTCs Induce the Pro-Tumorigenic Differentiation of PMN-MDSCs through Nodal-Cripto Axis

**Figure 8:**
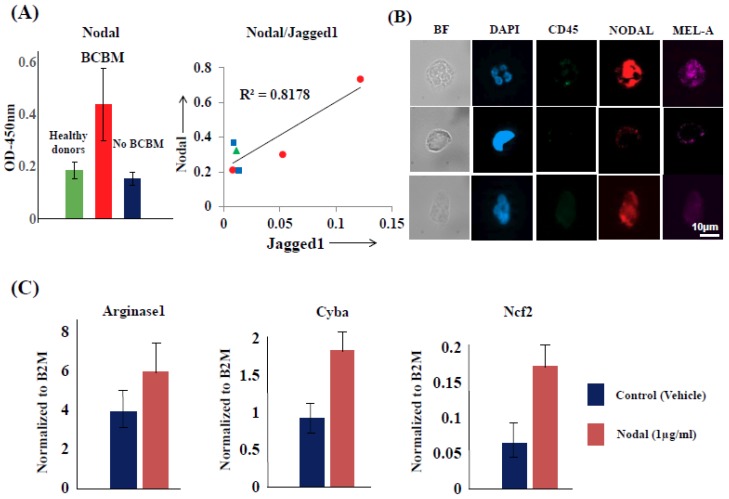
(**A**) Significant Nodal expression in serum of BCBM patients vs. patients with No BCBM but diagnosed with metastasis to other organs vs. healthy donors (Left panel). High correlation (R^2^ = 0.8178) between BCBM Nodal and PMN-MDSC Jagged1 expression in the same patient cohort type (Right panel). (**B**) Nodal expression positivity/heterogeneity in CTCs isolated from blood of melanoma patients by FACS. Staining was performed employing high-definition immunofluorescence (IF) microscopy, as previously described [3]. (**C**) Increased expression of Arginase1, Cyba, and Ncf2 gene expression by treatment of PMN-MDSCs with recombinant human Nodal (1 μg/mL) [35]. B2M = Beta 2 microglobin (control). Data shown are mean ± SD of four independent experiments (*n* = 4).

**Figure 9:**
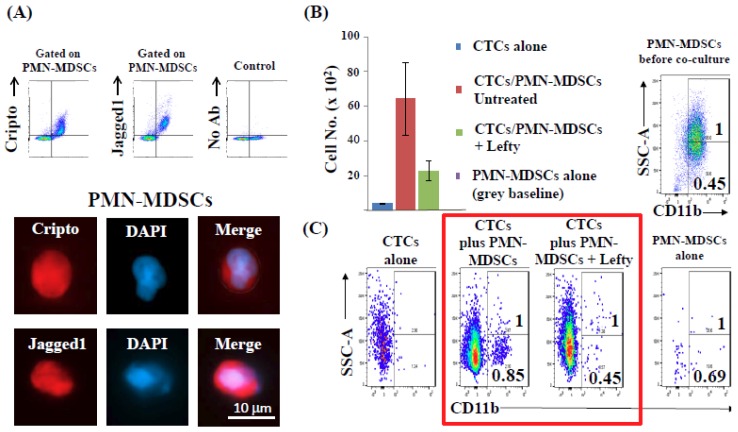
(**A**) Detection of Cripto/Jagged1 over-expression in PMN-MDSCs isolated from blood of BCBM patients by FACS followed by high-definition immunofluorescence (IF) microscopy. Single-cell images of a representative experiment (*n* = 4) are shown. (**B**) Co-cultures of CTCs with PMN-MDSCs isolated from BCBM patients not treated (red), or treated with Nodal inhibitor Lefty (green). CTCs (blue) or PMN-MDSCs cultured alone (grey, at baseline) represent negative controls. Data are mean ± SD of four independent experiments (*n* = 4). (**C**) Red frame shows PMN-MDSC transdifferentiation/phenotypic changes induced following co-culture with CTCs isolated from the same patient; however, changes were abrogated by incubation with Nodal inhibitor Lefty (1 µg/mL for 96 h at 37 °C) [34]. Ratios between CD11b+/SSC-A and CD11b+/SSC-A low are shown in a representative experiment (*n* = 4).

To investigate mechanisms by which CTCs modulate PMN-MDSCs towards a pro-tumorigenic phenotype, we used the Luminex platform (Luminex Corporation, Austin, TX, USA) to screen serum samples of metastatic breast cancer and melanoma patients, along with serum from healthy donors (controls). By employing a panel of cytokines reported to promote the “type 2” differentiation of PMN-MDSCs, we found that serum levels of Nodal, an embryonic morphogen involved in organ development and reactivated in aggressive melanoma and breast cancer [32,33], were significantly elevated in cancer patients. These findings correlated with both increased PMN-MDSC numbers as well as with patient’s brain metastasis burden vs. no brain metastasis (but diagnosis of metastasis elsewhere), vs. healthy donors (control) (Figure 8A). Second, we detected high expression of Nodal mRNA and Nodal protein in patient-derived CTCs at single-cell level (Figure 8B). Treatment of PMN-MDSCs with human recombinant Nodal in vitro induced high expression of CD11b and CD66b, heightened ROS production, and increased mRNA expression of *ARG1*, *CYBA* and *NCF2* (Figure 8C), all features pathognomonic of a PMN-MDSC “type 2” response. Because potential Nodal-mediated CTC/PMN-MDSC cross-talk requires the presence of Nodal receptors on PMN-MDSCs, we screened CTC/PMN-MDSC clusters for various Nodal receptors by qPCR and found that the Nodal-specific co-receptor Cripto1 (*CFC1B*) was highly expressed in PMN-MDSCs. Importantly, Nodal expression was validated at the protein level by flow cytometry and immunocytochemistry. High Cripto and Jagged1 expression were detected among PMN-MDSCs isolated from patients diagnosed with breast cancer brain metastasis (BCBM) by multiparametric flow cytometry and IF microscopy (Figure 9A). To validate the presence of a functional Nodal-Cripto axis between CTCs and PMN-MDSCs, we co-cultured PMN-MDSCs with Nodal-expressing tumor cells and in presence/absence of Nodal inhibitor Lefty [34]. We found that in the presence of Lefty not only numbers of CTCs from co-cultures with PMN-MDSCs were significantly reduced but also that Nodal-expressing tumor cells failed to induce “type 2” differentiation of PMN-MDSCs (Figure 9B,C). Nodal-mediated upregulation of Cyba and Ncf2 also induced ROS production by PMN-MDSCs (data not shown). These findings suggest that tumor cell-secreted Nodal may be central to the mutual activation cycle of CTC/PMN-MDSC clustering and signaling pathways regulation to promote CTC survival and proliferation.

## 3. Discussion

CTCs shed intermittently by primary and metastatic tumors represent the fundamental pre-requisites to originate either metastasis (CTC shed from primary tumors), or metastasis of metastasis (CTC shed from primary metastatic disease). However, despite their relevance, technologies have only recently been developed to successfully isolate the entire spectrum of CTCs from patients’ blood, characterize them according to biomarkers and signatures, and interrogate their properties [24]. This is compounded by the extraordinary rarity, fragility and heterogeneity of CTCs [24,36]. Furthermore, most CTCs are destined to die in the foreign environment of blood [3,35,37]. Investigations into genesis, functionalities, and metastatic competency of CTC clusters are therefore relevant because they can represent a key mechanism to regulate CTC survival by cross-talk of CTCs with circulatory cells of the immune system, thus bypassing immune surveillance [1,2]. Although CTC clusters represent a minority (2–4%) of the overall CTC population in blood, the probability of generating metastasis has been optimized to be 50 times higher than singular CTCs [1,2], and their presence was related to poor patient survival in many clinical investigations [1]. Notably, most studies have focused on the elucidation of biomarkers and pathways of homotypic CTC clusters [2,38], rather than deciphering heterotypic CTC clusters [26].

Myeloid-derived suppressor cells (MDSCs) are immature myeloid cells which attenuate the immune response during acute disease [4,5]. However, their high immunosuppressive activity has distinctly different effects when responding to chronic diseases such as cancer. The prolonged expansion of activated MDSCs establishes a tolerogenic microenvironment which promote metastasis [6]. The population of MDSCs in the circulation of cancer patients increased with progression of disease, including melanoma and breast cancer [7,8]. Accordingly, a correlation between increased numbers of circulating MDSCs and CTCs established during the distant-metastasis stage could be a consequence of the tumor-cell promoting activities of MDSCs. For example, a correlation between numbers of circulating MDSCs and FACS-isolated K-RASmutmRNA+ CTCs from patients with pancreatic cancer was reported, suggesting that the establishment of liver metastases may be supported by immunosuppression-dependent CTC survival in blood [39]. Furthermore, the association between neutrophils and CTCs driving cell-cycle progression within the bloodstream was recently reported, providing a rationale for targeting cellular interactions of heterotypic CTC clusters [26].

PMN-MDSCs are well known to promote tumor progression through two mechanisms: 1) the suppression of anti-tumor immune response mounted by T cells and natural killer cells; and 2) the direct crosstalk with tumor cells to foster tumor-initiating capabilities. However, despite the importance of the latter, mechanisms for orchestrating CTC/MDSC interactions in the circulation remain mostly unknown. In this study, we provide evidence for the presence of naïve, heterotypic CTC clusters which consist of CTCs and PMN-MDSCs isolated in parallel from two distinct cohorts: patients diagnosed with metastatic melanoma or breast cancer.

Capturing heterotypic cell interactions using the CellSearch^®^ CTC platform revealed the presence of relatively small CTC clusters (2–3 cells/cluster) which is in stark contrast to the much larger clusters (10–30 cells/cluster) obtainable by the two cell filtration technologies (Parsortix^®^, Creatv™) used here. Given the automated processing and exclusion criteria of the CellSearch^®^ platform, it is likely that larger, naïve heterotypic clusters composed of CTCs complexed with immune cells are rarely presented because of their CD45+/CD34+ or CD45+ cell staining for melanoma and breast cancer samples, respectively. It is interesting to note that previous studies reporting tumor CTC only (homotypic) clusters used an enrichment strategy that included CD45-depletion [2,28,38]. This may have prevented the detection of heterotypic clusters consisting of CTCs and immune cells because of their inadvertent exclusion during sample processing. We have shown previously that a distinct CD44+/CD24- sub-population of breast cancer CTCs is not detectable by CellSearch^®^ (“stem-like” CTCs) [3]. Similarly, our data show that another sub-population of CTCs, e.g., one implicated in heterotypic CTC/PMN-MDSC cluster formation, is not readily detectable.

We tested the hypothesis that circulatory PMN-MDSCs can directly interact with CTCs affecting their biology, thus “priming” CTCs as cluster-mediated, self-perpetrating cycle: the induction of pro-survival signaling pathways in CTCs via PMN-MDSC-generated ROS and the activation of Notch signaling pathway could be reciprocated by CTCs promoting PMN-MDSC pro-tumor functions via the secretion of CTC Nodal protein interacting with PMN-MDSC Nodal receptor Cripto and signaling. Interactions of patient-isolated CTCs/PMN-MDSCs lead to broad changes in CTC characteristics and behavior, inducing cell-cycle regulators involved in CTC survival, proliferation and metastatic potency. We discovered a novel Nodal-Notch1-Jagged1 signaling axis which can support these activities. Increased ROS produced by PMN-MDSCs upregulated Notch1 in CTCs through the ROS-NRF2-ARE axis, thus priming CTCs to respond to ligand-mediated, PMN-MDSC-driven Notch activation. Conversely, Notch1 pathway activation generated high Nodal expression and secretion affecting CTC/PMN-MDSC clustering and PMN-MDSC trans-differentiation/phenotypic changes.

**Figure 10:**
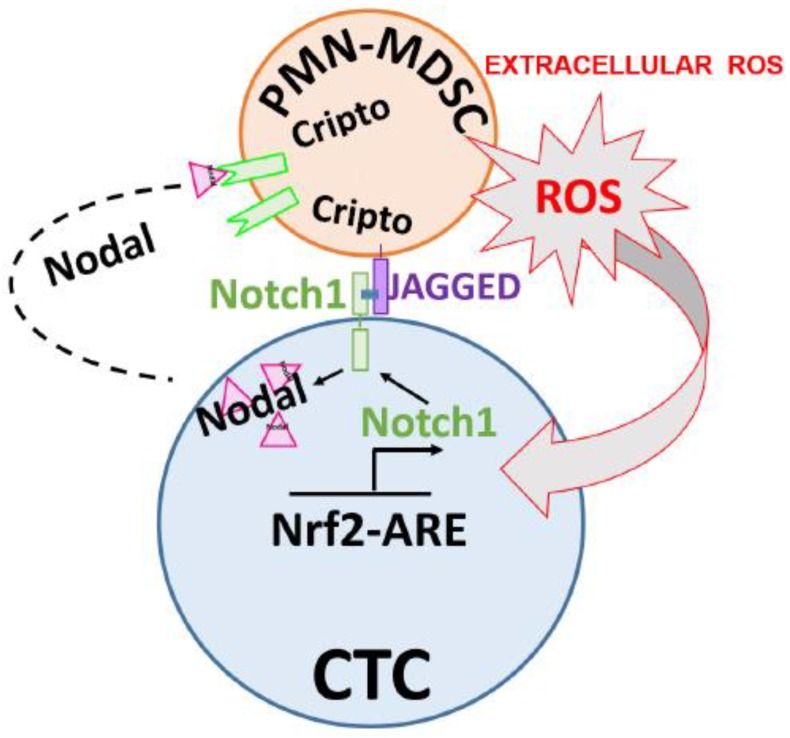
Model outlining biomarkers and pathways between CTCs and PMN-MDSCs interactions (CTC/PMN-MDSC cluster) isolated from blood of metastatic cancer patients. Large red arrow shows PMN-MDSC-secreted ROS affecting CTC-associated Nrf2/Notch1/Nodal pathway. Small black arrows refer to induction of CTC Nrf2-ARE, Notch1 and Nodal gene expression, respectively.

Notch ligands and Notch are cell-surface proteins, and their signaling is initiated when neighboring cells come into contact with each other. Furthermore, ROS species are short-lived and have a limited range of action. Therefore, CTC/PMN-MDSC clusters may present unique micro-niches in blood for initiation and effectiveness of CTC/PMN-MDSC crosstalk by the activation of Notch/ROS/Nodal signal circuitry as revealed in this study (Figure 10).

The precise biological contexts of CTC survival while circulating in blood remain largely unexplored. Only a small proportion of disseminated tumor cells are successful in establishing metastasis whereas most are eliminated in the circulation or persist at distant organs as dormant cells [3,35,37,40]. However, when aggregated in homotypic clusters, CTCs exhibit prolonged survival and decreased apoptosis, the consequence of which is an increase in metastatic potential [2,26,38,41]. Although investigations have demonstrated presence of CTC clusters that comprise non-tumor cells, e.g., neutrophils [26], this study is the first to demonstrate the detection and functionality of naïve, heterotypic clusters that consist of CTCs and circulatory PMN-MDSCs from cancer patients.

This study has some limitations. First, analyses were performed on a defined number of patients; therefore, these are initial observations which preclude conclusions that all patients with melanoma and breast cancer will follow these models and pathways. Second, we profiled a limited number of single CTCs and PMN-MDSCs obtained from heterotypic clusters dissociation, which, although done stochastically, may present inherent sampling bias. Third, because we did not perform inhibitory studies of these pathways in vivo affecting metastasis, we cannot rule out the possibility that the biomarkers we uncovered in CTCs and/or PMN-MDSCs could also potentially be associated with other cell types in vivo. Additional studies are needed to clarify these aspects. Regardless, the detection of heterotypic CTC/PMN-MDSC clusters in vivo and in vitro, the discovery of mutual activation crosstalk with specific interactions of CTCs with PMN-MDSCs, and the correlation between circulatory PMN-MDSCs and CTCs with distant metastasis warrant further investigation. This can be critical for improved understandings of immunosuppression-dependent mechanisms affecting CTC survival and actions as metastasis-competent cells.

## 4. Materials and Methods

### 4.1. Antibodies and Inhibitors

For multi-parametric flow cytometry, immunofluorescence/immunohistochemistry and DEPArray analyses, primary antibodies were obtained from the following sources: FITC-CD45 (#304054; 1:200), FITC-CD34 (#343504; 1:200), FITC-CD105 (#323204; 1:200), FITC-CD90 (#328108; 1:200), FITC-CD73 (#344016; 1:200), FITC HLA-A/B/C antibody (#311404; 1:200), PerCP/Cy5.5-CD146 (#342014; 1:100), PE-Human NG2/MCSP (#FAB2585P, 1:100), BV421-Ki67 (#350506; 1:100), were obtained from Biolegend, Inc. APC-Cy7-CD44 (#103028, 1:100) BV510-CD24 (#311126, 1:100), PE-Pan-Cytokeratin (#5075, 1:100) were purchased from Cell Signaling Technology, Inc. Anti-Mel-A antibody (# AC12-0297-03; 1:200) was obtained from Abcore, and FITC-Anti-S100 (#ab76749; 1:50) was purchased from Abcam. For immunohistochemistry, anti-human, anti-Mel-A antibody (# ab51061; 1:100), HLA-ABC (#565292; 1:100) were obtained from BD Biosciences, Inc. (San Jose, CA, USA). Anti-mouse secondary IgG anti-human antibodies used for IHC staining were received from Santa Cruz Biotechnology, Inc. Antibodies for immunofluorescence staining were obtained from Cell Signaling Technology, Inc. (1:500 dilution of stock solution), as previously described [3,22]. NRF2 inhibitor ML385 (#2114) was obtained from Cayman, Inc. while Nodal inhibitor Lefty (746-LF/CF) was purchased from R&D Systems, Inc. (Waltham, MA, USA).

### 4.2. Patient Blood Collection, PBMC Isolation, and Multi Parametric Flow Cytometry (FACS)

Melanoma and breast cancer patients were accrued according to protocols approved by the Institutional Ethical Review Boards at the University of Texas MD Anderson Cancer Center and Houston Methodist Research Institute (HMRI). All patient blood samples were collected after receiving informed written consent and according to the principles of the Declaration of Helsinki. Peripheral blood (18–20 mL) was obtained at the middle of vein puncture and was collected in CellSave^®^ tubes (Menarini Silicon Biosystems, Inc., Bologna, Italy), or EDTA tubes under aseptic conditions. Samples were sent immediately to the laboratory for CTC/PMN-MDSC isolation and analysis. All blood samples were processed within 24 h of collection, and were carefully utilized in experimental approaches, e.g., PBMCs were used in CTC/PMN-MDSC experiments and matched serum was used for extracellular ROS experiments. Since CTCs are present in the buffy coat fraction, they were enriched by red blood cell (RBC) lysis reagent using methodologies previously reported [3]. PBMCs were isolated by established procedures [3]. Briefly, whole blood was treated with red blood cell lysis buffer (154 mM NH_4_Cl, 10 mM KHCO_3_, 0.1 mM EDTA) at 1:25 ratio, followed by incubation at room temperature (25 °C) for 5 min, then pelleting the remaining blood cells at 300 g for 10 min. Mononucleated cell pellet was then washed twice with 1× PBS (with 5 mM EDTA) and used for fluorescence labeling followed by multi-parametric flow sorting (FACSAria™ II, BD Biosciences, San Jose, CA, USA), as previously described [3,18]. Forward scatter area vs. height was used for doublet discrimination and DAPI (impermeant to live cells with intact cell membrane) to determine cell viability. FITC was used as “dump” channel and FITC+ cells were eliminated from downstream analyses. Antibodies for specific markers to isolate breast cancer/melanoma CTCs or PMN-MDSCs were used as mentioned in main text. Data recorded during cell sorting were analyzed by DIVA acquisition software version 8.0.1 (BD Biosciences). Antibodies and reagents described above were used. Data generated by FACS were analyzed by FlowJo V10, as previously described [3].

### 4.3. CTC/PMN-MDSC Cluster Capture

7.5 mL peripheral whole blood collected in EDTA-coated tubes were loaded on a microfluidic chip (8 µm) within 8 h of blood draw. Flow protocols of microfluidic cell separation were employed according to guidelines and protocols for Parsortix^®^ or Creatv™ provided by respective Companies. Protocols were validated first using spike-in samples of Vybrant™ DiO-labeled melanoma (70W-SM3) and breast cancer (MDA-MB-231BR) cell lines in blood of healthy donors. For immunofluorescence (IF) staining, CTC/PMN-MDSC clusters after capture by the Parsortix^®^ chip were subjected to on-cassette-staining according to manufacturer’s guidelines, followed by fluorescence microscopy detection (10×–40× magnification). Cells designated as CTCs stained negative for CD45, positive for DAPI, positive for EpCAM/Pan-CK (breast cancer; CTC definition by CellSearch^®^) or negative for CD34 but positive for MelA/CD146 (melanoma), respectively. Cells designated as PMN-MDSCs stained negative for above tumor markers; however positive for DAPI, CD45, CD33, CD11b and CD15 markers. In addition, these cells displayed nuclear morphology typical of PMN-lineage. For CellSearch^®^ CTC analysis, patient peripheral blood was processed using CellTracks^®^ and CTC Melanoma Cell or Epithelial Cell (breast cancer) Kits (Menarini Silicon Biosystems, Inc., Bologna, Italy), following the manufacturer’s guidelines. For analyses involving murine blood, harvested blood (500 µL) was spiked with blood (7.0 mL) from healthy donors and was then processed by CellSearch^®^ for CTC enumeration/CTC clusters detection using the manufacturer’s guidelines.

### 4.4. Cell Culture and Cell Analyses

Early passage human breast cancer (MDA-MB-231BR) and melanoma (70W-SM3) brain metastatic variants of parental cell lines (MDA-MB-231P/70W, respectively) were cultured in DMEM/F12 (Invitrogen, Wltham, MA, USA) supplemented with 10% (*v*/*v*) FBS (Invitrogen) under prescribed conditions [42,43]. Cells were maintained at 37 °C in a humidified 5% CO_2_/95% air (*v*/*v*) atmosphere and passaged using trypsin-EDTA (10%-5 mM) before reaching confluency. Cells were STR-authenticated and assessed as pathogen-free by periodic testing for *Mycoplasma* contamination and in vivo abilities to generate brain metastasis (test every 20 passages). Luciferase-tagged MDA-MB-231BR/70W-SM3 cells (highly brain-metastatic variants of parental MDA-MB-231 and 70W cells) were obtained following methodologies previously reported [44]. Briefly, pQCXIB CMV/TO LUC (Addgene plasmid # 17475) was packaged in Phoenix Ampho cell lines (obtained from Tissue Culture Core, Baylor College of Medicine, Houston, TX, USA). Forty-eight hour supernatants were used to transduce respective cell lines. Cells were then selected with blasticidin (1 µg/mL), and luciferase activity was measured in single cell-derived clones. Clone with highest luciferase activity was used for subsequent experiments, allowing in vivo monitoring of cell location and cell number by IVIS. For mouse studies, luciferase labeled tumor cells were mixed with patient-derived PMN-MDSCs of matching cancer type, and cell mixture was administered to NSG mice via intra-cardiac injection. At various time points post-cell injection (10 min and afterwards), mice were monitored by intraperitoneal injection of d-luciferin and bioluminescence imaging by IVIS.

For DEPArray analyses of CTCs/PMN-MDSCs from co-cultures, clusters were retrieved upon 96 h of co-culturing conditions by CellCelector™, dispersed by brief Trypsin/EDTA treatment, and stained for markers specific for CTCs or PMN-MDSCs, followed by intracellular staining of Ki67. Stained samples were loaded on DEPArray for cell identification and enumeration of Ki67(+) cells, as previously described [3,18] and according to manufacturer’s guidelines and protocols (Menarini Silicon Biosystems, Inc., Bologna, Italy).

### 4.5. Live-Cell Microscopy

To evaluate proliferation of CTCs in co-culture with PMN-MDSCs, CTCs isolated by multiparametric flow cytometry from patient blood were labeled with cell tracker dye (Vybrant dye, green), then mixed with unlabeled PMN-MDSCs of the same patient and cultured in 96-well plates. Effects of CTC/PMN-MDSC interactions on CTC proliferation and cultures treatment were monitored using IncuCyte^®^ (Essen Instruments, Ann Arbor, MI, USA), a live-cell imaging system. CTC proliferation was measured by IncuCyte^®^ employing time-lapse image acquisition and kinetics processing to quantify CTC proliferation over time. Same number of dye-labeled CTCs per well were cultured alone as comparison. Labeled CTCs were detected and tracked using a sequence of Incucyte^®^ live cell images (one image/hour) for 96 h.

### 4.6. Experimental Animals and Mouse Models

All animal studies were approved by our Institutional Animal Care and Use Committee protocol (HMRI Code: IS00004851, 08/02/2018). Immunodeficient animal experiments were performed using 4- to 8-week-old NOD Cg-Prkdcscid Il2rgtm1 Wjl/SzJ (NSG) mice (The Jackson Laboratory, Bar Harbor, MA, USA). Flow-sorted Lin-negative/CTC-enriched cell population (melanoma CTCs) and PMN-MDSCs (1–5.0 × 10^3^ Lin- cell population, 5.0–25 × 10^5^ PMN-MDSCs) from patient blood were injected alone or in combination (optimized 1:500 ratio was used as physiologically relevant) in NSG mice through cell intracardiac injection under aseptic conditions [22]. The same conditions were applied for luciferase-tagged cell lines (Luc-MDA-MB-231BR, Luc-70W SM-3) although at higher numbers (0.5–1.0 × 10^6^ tumor cells/animal, plus PMN-MDSCs at above ratio). Xenograft mice were euthanized 6 months after injection with patient-isolated Lin-negative/CTC-enriched cell population (melanoma CTCs), or 4 weeks after injection with cell lines.

Approximately 800 to 900 µL blood was collected in EDTA tubes by cardiac puncture of anesthetized mice for longitudinal monitoring of CTC/PMN-MDSC clusters. Clusters were then collected using filtration devices and dissociated employing the CellCelector™ platform (ALS, Inc., Jena, Germany) and its robotic arm, followed by trypsin treatment. Humanized mice were generated at HMRI using four-week-old NOD.Cg-Prkdcscid Il2rgtm1Wjl/SzJ−/− immunodeficient mice (Jackson ImmunoResearch Laboratories), as described [29]. Briefly, mice were irradiated by sub-lethal dose of 1.1 Gy whole body irradiation and monitored daily for clinical signs of radiation exposure for 14 days. Sub-lethally irradiated mice exhibiting no clinical symptoms were used for engrafting CD34+ hematopoietic progenitor cells (1.0–2.0 × 10^5^ cells/animal) (Lonza, Inc., Basel, Switzerland) through intravenous tail vein injection. The humanized mice model was validated for engraftment of human hematopoietic stem cells over a 10- to 12-week period for the detection of human specific HLA-ABC, CD45, CD33, CD3, CD19, CD14, CD15, and CD11b-positive cells in mouse peripheral blood through multi-parametric FACS analyses. Successful humanization was monitored by flow cytometry four weeks after cell transplantation demonstrating the presence of human B220(+) (B-cells), CD3(+) (T cells) and CD11b(+) (myeloid lineage) cells. Experiments were then initiated by intracardiac transplantation of patient-derived Lin-negative/CTC-enriched cell population (melanoma CTCs) and PMN-MDSCs at 8–10 weeks post-cell transplantation.

### 4.7. Immunofluorescence and Immunohistochemistry

FACS-isolated CTCs were subjected to quick air-dry on Millennia™ 2000 adhesive glass slides (StatLab), and fixed with 4% paraformaldehyde [23]. Cells were permeabilized (0.05% Triton X-100 in 1× PBS) for 30 min, followed by 30 min incubation in blocking buffer (1% BSA + 1% normal goat serum in 1× PBS). Next, immunofluorescent cell staining was employed using selected primary and secondary antibodies. Magnified (100×) images were captured using Zeiss Axio Observer microscope Z1 (Carl Zeiss, Inc., Jena, Germany), and data were analyzed using Zeiss ZEN2 software. Harvested tissue was processed and stained for H&E and other immunohistochemistry markers by the research pathology core at HMRI [18]. Images were captured by using EVOS XL Cell Imaging System (ThermoFisher Scientific, Inc., Waltham, MA, USA). Immunohistochemistry images were taken and quantified by free-access ImageJ software. Reciprocal intensity (250-*y*) was measured by subtracting the mean intensity of the stained area (*y*) from the maximum (250) unstained white area intensity. Student *t*-test, *type2*, *paired 2* were used to calculate the difference in reciprocal intensity between two groups (inhibitors treated vs. untreated) for each protein [18].

### 4.8. Extracellular ROS Determination

Reactive oxygen species (ROS) assessment in patient serum was performed using the CellROX™ Deep Red Reagent (ThermoFisher Scientific, Inc.), which is a sensitive ROS sensor. In its reduced state, CellROX™ Deep Red reagent is non-fluorescent; however, it becomes brightly fluorescent when oxidized by ROS, e.g., hydrogen peroxide, hydroxyl radical and superoxide anion [5,8]. Upon receipt, patient blood samples were stored at 4 °C for 30 min to 1 h in an upright orientation to allow the plasma and cell fractions to separate. A 1 mL aliquot of plasma was then centrifuged at 2000× *g* for 15 min at 4 °C to isolate the serum. 90 µL of patient serum was combined with 10 µL of 50 µM CellROX™ Deep Red reagent in a black walled, flat bottom 96-well plates. Sample plates were protected from light and incubated for 15 min at room temperature (25 °C) on a plate shaker. Fluorescence was measured using a BioTek Synergy HTX with excitation/emission of 635/665 nm, respectively. ROS levels were calculated by subtracting background fluorescence using a no CellROX™ Deep Red reagent control (patient sample with no reagent) and further normalized by H_2_O_2_ (10 mM; positive control). ROS levels were measured in triplicate and within 48 h of blood draw.

### 4.9. Whole Exome Sequencing (WES)

DNA was purified from cells isolated by FACS using NucleoSpin^®^ XS kit (Macherey-Nagel, GmbH & Co., KG, Bethlehem, PA, USA). The Nextera Exome kit (Illumina, Inc., San Diego, CA, USA) was used to construct the sequencing libraries, and sequencing was performed by Illumina NextSeq 500 sequencer employing the high-output module of 150 bp paired reads, operated by the ncRNA Core at MD Anderson Cancer Center (Houston, TX, USA). To capture somatic mutations in CTC and immune cells, the mean coverage was set at 200×, much higher than the standard 100× for cancer genome. CD45+ - selected population served as a reference for somatic mutations. WES coverage was 75× for germline diploid genome sequencing. Sequencing reads were mapped to HG38 using BWA v0.7.17, followed by removing PCR duplicates and Bam recalibration. Somatic variant calling was done with Varscan V.2.3.9 [45], filtered and annotated. Circos data visualization was generated using R package [46]. Mutation signatures were computed according to methods previously described [47].

### 4.10. Sequence Mapping and Statistical Analyses

Raw whole exome sequencing (WES) reads were pre-processed using Cutadapt (v.1.15) to remove bases with quality scores <20 and adapter sequences [48]. Clean sequence reads were aligned to the human reference genome UCSC build GRC38 by Burrows-Wheeler Aligner (BWA) software (v.0.7.17) [49]. Picard (v.2.8.16) (https://broadinstitute.github.io/picard/) was used to remove PCR duplicates. To detect SNVs, we implemented a discovery pipeline based on GATK (v.3.8.0) to recalibrate the base qualities and realigned the sequence reads around micro indels to obtain more accurate quality scores and to ensure a better alignment in regions with micro indels. Then, Varscan (v.2.3.9) and Strelka (v.1.0.15) programs were used to detect critical somatic SNV and INDEL mutations, respectively. All variants were annotated by ANNOVAR RefSeq transcript annotation databases [48].

### 4.11. Transcriptome Analyses of CTCs Co-Cultured with PMN-MDSCs

Following isolation of CTCs and PMN-MDSCs from patient blood by flow cytometry sorting, cell populations of a given patient were cultured individually or mixed together to generate CTC/PMN-MDSC co-cultures in a sphere-forming medium on low-attachment plates [8]. After four days of co-culture, CTC/PMN-MDSC clusters were picked-up from the culture wells using the robotic micromanipulator device of the CellCelector™ (ALS, Inc., Jena, Germany). Clusters were dissociated by brief Trypsin/EDTA treatment, stained and submitted for DEPArray analyses [3]. CTCs and PMN-MDSCs were distinguished by their specific expression pattern of tumor cell markers vs. CD45, CD33 and CD11b MDSC markers. CTCs were recovered from the DEPArray cassette, and their gene expression was then analyzed by microarray (Human Clariom D, Affimetrix, Inc., Santa Clara, CA, USA) [18]. CTCs of cultures without PMN-MDSCs were subjected to the same DEPArray/Microarray procedure for comparison (controls). Gene expression differences between control and co-cultured CTCs were evaluated on the Transcriptome Analysis Console (TAC).

## Supplementary Materials

Supplementary materials can be found at https://www.mdpi.com/1422-0067/20/8/1916/s1.
