# Supplementary material for: PMN-MDSCs Enhance CTC Metastatic Properties through Reciprocal Interactions via ROS/Notch/Nodal Signaling
Source: Int J Mol Sci. 2019 Apr 18;20(8):1916. doi: 10.3390/ijms20081916 (PMC6514876; doi:10.3390/ijms20081916)
Supplement: Supplementary file 1 [file ijms-20-01916-s001.pdf]

International Journal of
Molecular Sciences

MDPI

Primary Cancer	Metastasis	HER-2 Status	ER/PR Status	BRCA1/2 Status	Stage at diagnosis	Ki67 Status
Breast, brain mets unknown	Node, liver, lung, bone	Pos	Pos/Pos	Not listed	4	Pos
Breast	Bone, brain, liver, node, mastectomy	Neg	Pos/Neg	Wild type	4	Pos
Breast	Lung	Neg	Neg/Neg	Neg		
Breast	Bone, Lung, Nodes	Neg	Pos/Weakly Pos	Wild type	T1cN0M0	Not listed
Breast, brain mets unknown	Node, liver, lung, bone	Pos	Pos/Pos	Not listed	4	Pos
Breast Mastectomy	Nodes, liver, bone	Neg	Neg/Neg	Not listed	4	Pos
Breast	Bones	Neg	Pos/Pos	Neg		
Brain Mets unknown, MASTECTOMY	Node, axilla, left posterior pleura, skin	Neg	Neg/Neg	Neg	pT1N0M0	Not listed
Breast Brain Mets unknown, MASTECTOMY	Node, axilla, left posterior pleura, skin	Neg	Neg/Neg	Neg	pT1N0M0	Not listed
Breast	liver, bone, node, lung	Neg	Pos/Pos	BRCA 1 & 2	4	Not listed
Breast Mastectomy	mets: liver and node	Neg	Neg/Neg	Wild type	T2N0M0	Pos
Breast Mastectomy	mets: liver and node	Neg	Neg/Neg	Wild type	T2N0M0	Pos
Breast Mastectomy	Node, lung, bone	Neg	Weak Pos/Neg	Wild type	T4dN2aM0	Pos
Breast Mastectomy	Lung, node	Neg	Neg/Neg	Wild type	4	Not listed
Breast Mastectomy	Lung, node	Equivocal	Neg/Neg	Not listed	T2N0MO	Pos

Figure S1. Breat cancer patients' clinical-pathological parameters.

Gender	Age	Type of primary	If brain mets – brain as 1st site of metastatic presentation	Stage	Mutation Status	Site of disease at time of sampling	Hx or current Parenchymal brain mets	Previous Therapies	Current Therapy at time of blood draw	Disease status at time of blood draw
male	75	N/A	concurrent	IV M1c	BRAF 597S	none	yes	ipi/nivo	nivo	currently in complete remission
female	37	cutaneous	yes, with other sites	IV M1c	BRAF V600E	brain, liver	yes	dabrafenib/trametinib, pembro,	pembro, dabrafenib/trametnib	10/2017-progression in brain and body
Male	61	cutaneous	No	IV M1b	BRAF V600R	lung	no	nivo	dabrafenib/trametinib	stable
female	30	cutaneous	no	IIIc	TERTm p53, CDk4, BRAFV600E, BRCA2	NED	no	none	none	NED
male	45	cutaneous	no	IVM1a	BRAF V600E	LN	no	none	dabrafenib/trametinib	improving
female	48	cutaneous	yes	IV M1c	unknown	brain, lung	yes	adjuvant GMCSF	none	progressing
male	79	mucosal	no	IV M1b	KIT	lung, LN	no	ipi and nivo	nivo	responding
male	69	NA	yes	IV M1d	BRAF wildtype	brain, LMD, lung, abdomen/peritoneum/omentum/visceral	yes	pembro	none	progressing
male	41	NA	no	IIIc	NRAS, RB1, p53	NED	no	neoadjuvant nivo	none	NED
male	68	cutaneous	no	IIIc	BRAF V600E	SQ	no	pembro	pembro	responding
female	61	cutaneous	no	IV M1c	no BRAF	SQ, lung, LN	no	None	none	progressing and treatment naive
female	48	cutaneous	yes	IV M1c	unknown	brain, lung	yes	adjuvant GMCSF	none	progressing
male	69	cutaneous	yes	IV M1c	NRAS, CDKN2a, KIT	brain, SQ, ST/muscle, lung, liver, LN, abdomen/peritoneum/omentum/visceral	yes	none	none	progressing
male	45	cutaneous	no	IVM1a	BRAF V600E	LN	no	none	dabrafenib/trametinib	improving
female	30	cutaneous	no	IIIc	TERTm p53, CDk4, BRAFV600E, BRCA2	NED	no	none	none	NED
female	42	cutaneous	no	IIIa	unknown	NED	no	none	none	NED
Male	49	cutaneous	No	IV M1c	BRAF V600E	brain, lung	no	Polynoma, Ipi, Dabrafenib/Trametinib	Ipi/Nivo	worsening brain mets, worsening extracranial
male	54	cutaneous	no	IVM1c	BRAF V600E, CDKN2A	brain	yes	PV10	none	new brain mets
female	44	cutaneous	NA	IIIb	BRAF V600E, KDR, MAP2K1	NED	no	none	none	NED

Figure S2. Melanoma patients' clinical-pathogical parameters.

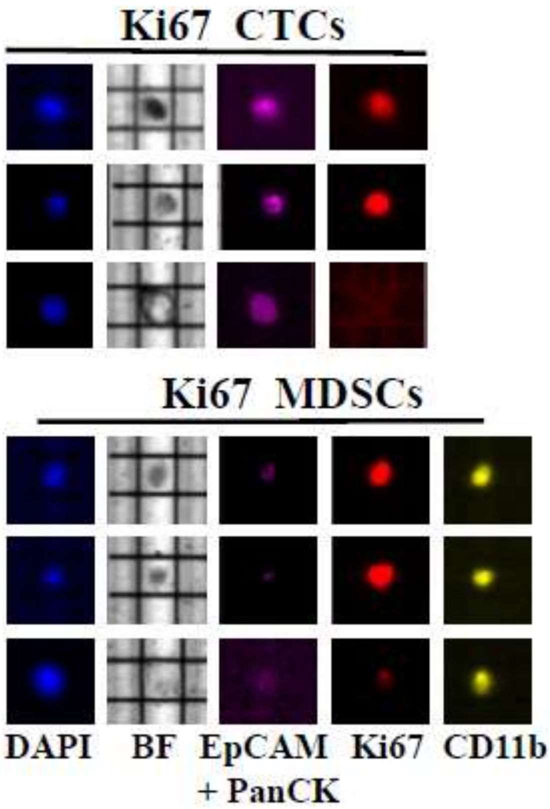

Figure S3. DEPArray analyses of CTCs and MDSCs dissociated from co-cultures and stained for respective biomarkers and Ki67. Ki67-positive cells represented 75% of all CTCs and 66% of all MDSCs, respectively.

© 2019 by the authors. Licensee MDPI, Basel, Switzerland. This article is an open access article distributed under the terms and conditions of the Creative Commons Attribution (CC BY) license (http://creativecommons.org/licenses/by/4.0/).
